# Essential Thrombocythemia among Patients with Myeloproliferative Neoplasms in Haematology Unit of a Tertiary Care Centre: A Descriptive Cross-sectional Study

**DOI:** 10.31729/jnma.7359

**Published:** 2022-04-30

**Authors:** Sanjit Kumar Sah, Sangam Shah, Sansar Babu Tiwari, Basanta Sharma Paudel, Biranmol Singh, Prakash Sharma, Sunil Sharma Acharya, Hritik Murarka, Sabin Thapaliya, Anjan Shrestha

**Affiliations:** 1 Maharajgunj Medical Campus, Institute of Medicine, Tribhuvan University, Maharajgunj, Nepal; 2 Department of Pathology, Maharajgunj Medical Campus, Institute of Medicine, Tribhuvan University, Maharajgunj, Nepal; 3 Department of Internal Medicine, Maharajgunj Medical Campus, Institute of Medicine, Tribhuvan University, Maharajgunj, Nepal

**Keywords:** *essential thrombocythemia*, *hematology*, *mutation*

## Abstract

**Introduction:**

Essential thrombocythemia, a myeloproliferative condition with an increased number of circulating platelets, is a rare hematological malignancy. The aim of the study is to find out the prevalence of essential thrombocythemia among patients with myeloproliferative neoplasms presenting in haematology unit of a tertiary care centre.

**Methods:**

This was a descriptive cross-sectional study at a tertiary care centre from September, 2020 to September, 2021 (Reference number: 48 (6-11) E2077/076). All the patients with a diagnosis of essential thrombocythemia and willing to give consent were included in the study while the patients with incomplete investigations were excluded. A sample size of 72 patients was taken and convenience sampling was done. Data were entered in Microsoft Excel 2010 and analysis was done by the Statistical Package for the Social Sciences Version 22.0. Point estimate at 95% Confidence Interval was calculated along with frequency and proportion for binary data along with mean and standard deviation for continuous data.

**Results:**

Among 72 patients with myeloproliferative neoplasms, the prevalence of essential thrombocythemia was found to be 17 (23.61%) (13.80-33.42 at a 95% Confidence Interval). The mean age of patients was 55.41+11.20 years with a male to female ratio of 9:8. The mean hemoglobin level and platelet count in patients were found to be 11.20+2.1 g/dl and 677000+262067.70 cells/mm^3^. Twelve (70.58%) of total patients were under low risk of essential thrombocythemia while 3 (17.64%) of them were at high risk.

**Conclusions:**

The prevalence of essential thrombocythemia was similar to other studies done in similar settings.

## INTRODUCTION

Essential thrombocythemia (ET), a myeloproliferative condition with an increased number of circulating platelets, is a rare hematological malignancy with an incidence of 0.38-1.7 per 100,000 per year.^1^ Females have twice more prevalence than males. It's diagnosis is based on the criteria that includes platelets >450x10^9^/l, presence of an acquired pathogenic mutation of Janus kinase-2 (JAK2)/ Calreticulin (CALR)/ Myeloproliferative leukemia (MPL), exclusion of other

myeloid malignancy, exclusion of reactive cause for thrombocytosis, normal iron stores and increased megakaryocytes in bonemarrow aspirate and biopsy.^2^

In the context of underdeveloped nations like Nepal, there is no specific research addressing the incidence and prevalence of Philadelphia chromosome (Ph)-negative myeloproliferative neoplasms.

The aim of the study is to find out the prevalence of essential thrombocythemia among patients with myeloproliferative neoplasms presenting to the haematology unit of a tertiary care centre.

## METHODS

This was a descriptive cross-sectional study carried out in Tribhuvan University Teaching Hospital (TUTH) of Kathmandu valley from September, 2020 to September, 2021 among 72 patients with myeloproliferative neoplasms. Ethical approval was obtained from the research ethics committee of the Institutional Review Committee (IRC) of the Institute of Medicine (IOM) (Reference number: 48 (6-11) E2077/076). Informed consent was obtained from all study participants to allow the use of anonymous personal and clinical data in research. Confidentiality of the information was maintained thoroughly by deidentification. Convenience sampling was done.

The sample size was calculated using the formula:

n = (Z^2^ × p × q) / e^2^

  = (1.96^2^ × 0.0253 × 0.9747) / 0.04^2^

  = 60

Where,

n = minimum required sample size
Z = 1.96 at 95% Confidence Interval (CI)
p = prevalence of essential thrombocythemia, 2.53%^3^
q = 1-p
e = margin of error, 4%

After adding a 10% non-response rate, sample size of 66 was obtained. However, 72 patients with myeloproliferative neoplasm were taken for the study. All the patients who presented with myeloproliferative neoplasms above 18 years of age were included in the study. Patients who did not have sufficient information on all of the findings were excluded from the study. Patients who did not have reports of a mutation test were also excluded.

Basic data including age, gender, occupation, nature, duration of symptoms, and other relevant history was taken from all the study participants in the study. The diagnosis of essential thrombocythemia was made in patients who fulfilled criteria 1-3 or criterion 1 plus criteria 3-5:^2^

1. Sustained platelet count ≥450 × 10^9^/l.
2. Presence of an acquired pathogenic mutation (JAK2, CALR or MPL genes)
3. No other myeloid malignancy (polycythemia vera, chronic myeloid leukemia, myelodysplastic syndrome or primary myelofibrosis)
4. No reactive cause for thrombocytosis and iron stores normal.
5. Bone marrow aspirate and trephine biopsy showing an increased megakaryocyte numbers (spectrum of morphology with predominant large megakaryocytes with hyperlobated nuclei and abundant cytoplasm).

The data were collected in the proforma. Data were compiled, edited, and checked daily to maintain consistency. The data were entered in Microsoft Excel 2010. For statistical analysis, the Statistical Package for the Social Sciences (SPSS) Version 22.0 was used. Point estimate at 95% Confidence Interval was calculated along with frequency and proportion for binary data along with mean and standard deviation for continuous data.

## RESULTS

Among 72 patients with myeloproliferative neoplasms, the prevalence of ET was found to be 17 (23.61%) (13.8033.42 at a 95% Confidence Interval). The mean age of patients was 55.41±11.20 years. Out of 17 patients, 9 (52.94%) of them were female while 8 (47.05%) were males. Nine (52.94%) of the patients were in service and 7 (41.17%) had business. All of them ie 17 (100%) were married and belonged to the middle-class families in the hilly belts of Nepal (Table 1).

**Table 1:** Demographic details (n= 17).

Variables	n (%)
**Sex**	
Male	8 (47.05)
Female	9 (52.94)
**Ethnicity**	
Brahmin	8 (47.05)
Chhetri	3 (17.64)
Janajati	4 (23.52)
Dalit	1 (5.88)
Others	1 (5.88)
**Religion**	
Hindu	13 (76.47)
Buddhist	9 (52.94)
**Occupation**	
Business	7 (41.17)
Work	9 (52.94)
Others	1 (5.88)

None of the patient had essential thrombocythemia in their family members. The arterial and venous thrombosis was seen in 8 (47.05%) and 5 (29.41%) of the patients respectively. Nine (52.94%) patients smoked and 8 (47.05%) consumed alcohol. Only 1 (5.88%) patient consumed a vegetarian diet. Out of 17 patients, 9 (52.94%) had pallor while 3 (17.64%) of the patients presented with edema. Only 1 (5.9%) patient presented with cyanosis. Six (35.29%) had hepatomegaly only and 6 (35.29%) had splenomegaly only (Table 2).

**Table 2:** Clinical features and examination findings (n= 17).

Symptoms	n (%)
Visual disturbance	6 (35.29)
Weight loss	12 (70.58)
Fever	7 (41.17)
Bleeding episode	8 (47.05)
Fatigue	14 (82.35)
Bone pain	7 (41.17)
Headache	14 (82.35)
Dizziness	7 (41.17)
**Thrombosis**	
Arterial thrombosis	8 (47.05)
Venous thrombosis	5 (29.41)
**Comorbidities**	
Hypertension	6 (35.29)
Diabetes mellitus	3 (17.64)
Chemotherapy for solid tumors	2 (11.76)
TB	4 (23.52)
Surgery	2 (11.76)
HIV/Hep B/Hep C	1 (5.88)
**Personal**	
Smoking	9 (52.94)
Alcohol	8 (47.05)
Vegetarian	1 (5.88)
Family history of ET	-
**Examinations**	
Pallor	9 (52.94)
Icterus	-
Clubbing	-
Cyanosis	1 (5.88)
Edema	3 (17.64)
**Abdomen findings**	
Hepatomegaly	6 (35.29)
Splenomegaly	6 (35.29)
Hepatosplenomegaly	4 (23.52)

Out of 17 patients, 6 (35.29%) had hypertension while 3 (17.64%) of them had diabetes mellitus. Similarly, 2 (11.76%) patients had a history of chemotherapy for solid tumors in the past and 4 (23.52%) of the patients suffered from tuberculosis in the past. One of the patients had hepatitis B as one of the major comorbidities while 2 (11.76%) patients had surgery in the past.

The mean hemoglobin level in patients was found to be 11.20±2.10 g/dl while the total leukocyte count was 13266 cells/mm^3^. The mean platelets count in patients with ET was found to be 677000.00±262067.70 cells/ mm^3^. The average albumin and total bilirubin level in patients were 38.30 g/l and 6.50 mg/dl respectively. Similarly, the mean prothrombin time (PT) was 16.00±1.30 seconds while the mean level of urea was found to be 10.70±4.80 mmol/l (Table 3).

**Table 3:** Laboratory investigations of patients with Essential thrombocythemia (n= 17).

Laboratory parameters	(Mean±SD)
Hemoglobin (g/dl)	11.20±2.10
TLC (cells/mm^3^)	13265.80±13293.40
Neutrophils (%)	69.20±7.40
Lymphocytes (%)	23.60±6.90
Monocytes (%)	4.60±1.60
Platelet (cells/mm^3^)	677000±262067.70
ESR (in the 1st hour)	0.60±0.80
Total bilirubin (mg/dl)	6.50±1.90
AST (U/l)	31±8.30
ALT (U/l)	24.90±4.40
ALP (U/l)	158.20±85.20
Albumin (g/l)	38.30±6.20
PT (sec)	16±1.30
Urea (mmol/l)	10.70±4.80
Creatinine (mmol/l)	16.80±21.80
Sodium (meq/l)	140.20±3.30
Potassium (meq/l)	4.70±0.40
Reticulocyte (%)	3.90±1.90

Most of the patients had JAK2 V617 mutation. Fifteen (88.23%) of the patients had JAK2 V617 mutation. Similarly, CALR exon 9 mutation was seen in 2 (11.76%) of the patients. The cases of MPL exon 10 were not reported in our study. Out of 17 patients, 14 (82.35%) had hypercellular marrow with megakaryocyte proliferation while 3 (17.64%) of them had normal cellularity. In 14 (82.35%) patients with hypercellular marrow showed matured megakaryocytes with hyper-lobulated nuclei (Table 4).

**Table 4:** Mutation and bone marrow of patients with essential thrombocythemia (n= 17).

Parameters	n (%)
**Mutation**	
JAK2V617	15 (88.23)
CALR exon 9	2 (11.76)
MPL exon 10	-
**Bone marrow**	
Megakaryocyte proliferation	14 (82.35)
Normal	3 (17.64)

On the basis of International Prognostic Score of Thrombosis by World Health Organization (WHO), 12 (70.58%) of total patients were under low risk while 3 (17.64%) of them were under high risk. One (5.88%) patient was under very low risk (Table 5).

**Table 5:** The international thrombosis (n= 17).

**Risk**	
High risk	3 (17.64)
Intermediate risk	1 (5.88)
Low risk	12 (70.58)
Very low risk	1 (5.88)

Two (11.76%) patients developed stroke as complications among the 17 patients during the treatment.

## DISCUSSION

Essential thrombocythemia (ET) is a kind of clonal myeloproliferative neoplasm (MPN) marked by unexplained platelet augmentation, overt thromboembolism, clinical bleeding, and, in rare cases, myelofibrosis or leukemia.^4^ It is an uncommon indolent malignancy of hemopoietic stem cells that are acquired and lacks the Philadelphia chromosome (Ph).^5^ Clinical characteristics, hematological markers, metabolic profile, and risk stratification in ET patients were all examined in this study. Due to asymptomatic clinical presentation, poor diagnostic facilities, and a tendency for late referrals, this hematological condition is uncommon in Nepal. Even the prevalence and incidence of this MPN are not known. To the best of our knowledge, this is the first study from Nepal.

ET is frequently identified as a disease of advanced age.^6^ The majority of the patients in our study were in their fifth or sixth decade of life. In major regional research conducted in India, the average age of ET patients was 55.41 years.^7^ When compared to previous international data, our findings contradict studies from Sweden and the United States, where the median age was 65-70 years.^8,9^ Perhaps the evident genetic differences between two racial groupings, as well as the greater average age in Western countries, explain this disparity. Despite the fact that ET had an impact on a relatively younger population in our study, the incidence of ET in females was greater than as well as identical to that seen in worldwide and regional studies.^10-12^

The majority of our patients (82.4%) presented with the symptoms of fatigue. This finding is different from the study of Kuwait and Thailand where 57.1% and 54.2% of the patient were asymptomatic respectively.^11-13^ Despite this, 11.76% of the cases in our study were asymptomatic. According to a study, 19% of the patients suffered erythromelalgia, 14.1% had thrombotic events, and 4.5% had bleeding.^13^ The most likely reason for the low number of asymptomatic patients is that they only come to the clinic when they acquire symptoms in our settings. Splenomegaly has been documented in 25% to 50% of ET patients.^14^ A study on the other hand, reported splenomegaly in 19% of their patients, which is lesser in comparison to our findings (35.3%). Another large series from Thailand found splenomegaly and hepatomegaly in 10.8% and 8.4% of the population, respectively.^11^ In our study, the average platelet count was 677000±262067.70 cells/ mm^3^.^7^ In agreement with our findings, studies from China and Thailand had also found similar findings.^11,15^

Risk stratification is effective for determining the degree of an illness and predicting the possibility of consequences. In a previous study in the United States, only 17% were considered to be at high risk.^16^ Comparable to data from developed countries, our study showed that the majority of patients (70.6%) were at low risk. The majority of the patient (88.2%) included in our study had JAK2V617 mutation while none of the patients were tested positive for MPL exon 10. The lack of an adequate number of samples in the study population may be the reason. Platelet count was relatively high in patients with homozygous JAK2 mutation and presented with more severe complications than that in cases without mutation. JAK2 mutation proved to render the progenitor thrombopoietin-independent which ultimately causes hyperproliferation of megakaryocytes leading to hypercellularity.

The sample size of our study was small. COVID-19 pandemic also caused a delay in the study because patients were unable to come for visits owing to the lockdown. Larger sample size would be a better predictor of our population's clinical and laboratory characteristics. The patients are still being followed-up. Hence, a long-term follow-up study assessing the outcome would be better in near future. This study was done at a single centre which might have led to selective bias. Despite these limitations mentioned above; this is the first study from Nepal.

## CONCLUSIONS

The prevalence of essential thrombocythemia from our study was similar to other studies similar done in similar settings. The study revealed that ET is uncommon in Nepal. Patients usually present when the symptoms are developed unlike others where most patients are asymptomatic. Prospective trials on large patient series should be undertaken to examine the disease spectrum further, and novel prognostic molecular tests should be included.
